# Idiopathic male infertility and polymorphisms in the DNA methyltransferase genes involved in epigenetic marking

**DOI:** 10.1038/s41598-017-11636-9

**Published:** 2017-09-11

**Authors:** Qiuqin Tang, Yiqiu Chen, Wei Wu, Hongjuan Ding, Yankai Xia, Daozhen Chen, Xinru Wang

**Affiliations:** 1 State Key Laboratory of Reproductive Medicine, Department of Obstetrics, Nanjing Maternity and Child Health Hospital, Obstetrics and Gynecology Hospital Affiliated to Nanjing Medical University, Nanjing, 210004 China; 2 State Key Laboratory of Reproductive Medicine, Institute of Applied Toxicology, School of Public Health, Nanjing Medical University, Nanjing, 211166 China; 3 Key Laboratory of Modern Toxicology of Ministry of Education, Nanjing Medical University, Nanjing, 211166 China; 4 Clinical Laboratory, Wuxi Maternity and Child Health Care Hospital Affiliated to Nanjing Medical University, Wuxi, 214002 China

## Abstract

The purpose of this study was to investigate the association between male infertility and single-nucleotide polymorphisms (SNPs) of DNA methyltransferases (DNMT) genes (*DNMT3B*: rs2424909, *DNMT1*: rs4804490, *DNMT3A*: rs1550117 and *DNMT3L*: rs7354779). Eight hundred and thirty three idiopathic infertile males and four hundred and ten fertile controls from the hospitals affiliated to Nanjing Medical University between 2010 and 2012 were recruited in the study. We demonstrated a significantly increased risk of idiopathic infertility with abnormal semen parameters in association with the heterozygous genotype of variant rs4804490. Moreover, the AA genotype of variant rs4804490 was associated with significantly decreased risk for male infertility with abnormal semen parameters. A decreased risk of idiopathic infertility with abnormal semen parameters was associated with the homozygous genotype of variant rs2424909. These results suggested that variants in different *DNMT* genes have different relationships with idiopathic male infertility, and Chinese men carrying these variants have an increased or decreased risk of abnormal semen parameters.

## Introduction

Male infertility is a heterogeneous disorder that contributes to the impairment of spermatogenesis. Clinical investigations have confirmed that a significant proportion of male infertility is idiopathic, the molecular mechanisms underlying the defects have not been elucidated^1, 2^. Most studies published to date support the view that abnormal epigenetic changes also resulted in male infertility. Lande-Diner and other researchers have concluded that DNA methylation is an essential epigenetic modification specifically related to gene silencing^3^. Aberrant DNA methylation has been proposed as a possible mechanism compromising male fertility^4–6^. The aberrant DNA methylation found in infertile males may be a consequence of a dysfunction in the machinery involved in establishing and maintaining normal DNA methylation. Accumulating evidence has indicated that male infertility results from gene mutations and single-nucleotide polymorphisms (SNP)^7, 8^.

Mouse models have shown that aberrant DNA methylation patterns resulting from gene targeting of the *DNMTs* may cause spermatogenic defects^9–12^. DNA methyltransferases (DNMTs) are a family of proteins responsible for transferring methyl groups specifically to cytosine in CpG dinucleotides^13, 14^. Genes involved in the epigenetic pathways are likely to affect DNA methylation and are associated with male infertility. Researchers have analyzed the genetic variants within the genes to investigate the epigenetic processes in the aetiology of diseases^15^.

Four DNMTs (*DNMT1* ^16^, *DNMT3A*, *DNMT3B* ^17–19^, and *DNMT3L* ^20, 21^) have been extensively characterized in mammals. *DNMT1*, which is often referred to as the maintenance methyltransferase, is responsible for maintaining pre-existing methylation patterns during DNA replication^22, 23^. *DNMT3A* and *DNMT3B* are considered to be de novo DNA methyltranferases, which are critical in the dynamic DNA methylation process during embryogenesis and pathogenesis^24^. In addition to these active enzymes, the DNMT3L protein, catalytically inactive by itself, also contributes to de novo methylation by interacting with the catalytic domains of *DNMT3A* and *DNMT3B* and enhancing their enzymatic activity^25–27^. DNA methylation, mediated by DNMTs, is required for proper embryonic development^18, 28^ and for the formation of mature functional germ cells^9, 10, 29^. Dysregulation of the DNMTs may lead to various conditions^30^, including spermatogenic defects^12^, autoimmune disorders^31^ and cancer^13, 32^. To better understand the relationships between polymorphisms in *DNMT*s and idiopathic male infertility, we studied the associations between genetic variation in *DNMT* genes and altered semen parameters. We show that variants in the *DNMT3B* and *DNMT1* may have different relationships with idiopathic male infertility. Furthermore, we compared the transcriptional factors between C allele and T allele of rs2424909, C allele and the A allele of rs4804490.

## Materials and Methods

### Subject recruitment and sample collection

The study was approved by the Institutional Ethics Committee of Nanjing Medical University. All activities involving human subjects were done under full compliance with government policies and the Helsinki Declaration. The participants wrote informed consents prior to the study. All study subjects were consecutively recruited from the Affiliated Hospitals of Nanjing Medical University between 2010 and 2012 (NJMU Infertility Study). The inclusion criteria consisted of five terms: (i) a normal 46, XY karyotype; (ii) absence of Y chromosomal microdeletions of AZF region; (iii) lack of hypogonadotropic hypogonadism; (iv) normal sexual and ejaculatory functions and no seminal tract obstruction, varicocele; (v) no history of infection or other diseases that could affect fertility. Finally, 833 men are included as the study subjects. The controls were healthy men who had fathered at least one healthy child, without assisted reproductive measures during the same period as those of the cases recruited in the same hospital. A scheduled interview was arranged for each subject to collect the basic information, including personal background, lifestyle factors, occupational and environmental exposures, sexual and reproduction status, genetic risk factors, medical history and physical activity (e.g. exercise status). Then, each subject donated a 5-ml peripheral blood sample and a semen sample for genetic testing. These patients and healthy donors were all ethnically Han Chinese east China.

### Semen analysis

Semen samples were obtained in private by masturbation into a sterile wide-mouth and metal-free glass container after a recommended at least 3-day sexual abstinence. After liquefaction at 37 °C for 30 minutes, conventional semen analysis was conducted in accordance with guidelines of the WHO Laboratory Manual for the Examination of Human Semen, including semen volume, sperm number per ejaculum, sperm concentration, motility, progression and motion parameters by using Micro-cell slide and computer-aided semen analysis (CASA, WLJY 9000, Weili New Century Science and Tech Dev.). Each sample was assessed twice with strict quality control.

### Variant selection and genotyping

Genomic DNA was extracted from leukocyte pellets of the venous blood by traditional proteinase K digestion, followed by phenol-chloroform extraction and ethanol precipitation. Genotypes were detected by the TaqMan allelic discrimination Assay on an ABI 7900HT real-time PCR system (Applied Biosystems, the United States). The gene variants studied were: *DNMT1* (rs4804490); *DNMT3A* (rs1550117); *DNMT3B* (rs2424909); *DNMT3L* (rs7354779). We selected only variants where the frequency of the minor allele was greater than 5%. For quality control, all genotyping assays were performed without knowing the case or control status of the subjects, and a random 5% of cases and controls were genotyped twice by different individuals.

### Predicting the association of variant *DNMT3B* rs2424909, *DNMT1* rs4804490, *DNMT3A* rs1550117 and *DNMT3L* rs7354779 and the binding sites of transcription factors

AliBaba2 is a program for predicting binding sites of transcription factors. We used the program to compare putative transcriptional factor-binding sites of rs2424909 and rs4804490, rs1550117 and rs7354779. Firstly, we searched the base sequences of rs2424909, rs4804490, rs1550117 and rs7354779 (51 bases). Then, by respectively entering C allele and T allele of rs2424909, C allele and A allele of rs4804490, A allele and G allele of rs1550117, C allele and T allele of rs7354779, the different binding sites of transcription factors emerged.

### Statistical analyses

Semen parameters were dichotomized based on WHO reference values (World Health Organization, 1999) for semen volume (<2 ml), sperm concentration (<20 × 10^6^/ml), sperm number per ejaculate (<40 × 10^6^/ml) and sperm motility (<50% motile sperm). The control group consisted of proven fertile men with all four semen parameters at or above the WHO reference value. The subjects with idiopathic infertility were first divided into two groups according to semen parameters: case Group I was defined as idiopathic infertile men with all four semen parameters at or above the WHO reference value and the subjects in case Group II had at least one of the abnormal semen parameters. Case Group II was divided into four subgroups: Subgroup I (semen volume <2 ml), Subgroup II (sperm concentration <20 × 10^6^/ml), Subgroup III (sperm number per ejaculate <40 × 10^6^/ml) and Subgroup IV (sperm motility <50% motile sperm). Analysis of variance was used to compare the mean age, body mass index (BMI) and pack-years of cigarette smoking between case and control groups. The χ^2^ test was used to evaluate the difference in smoking status and alcohol status between the two groups. Multivariate logistic regression analysis was performed to obtain the odds ratios (OR) for male infertility and 95% confidence intervals (95% CI) with adjustment for age, BMI, smoking status and alcohol drinking. All statistical analyses were carried out using Stata (Version 9.0, StataCorp LP, TX, USA), and *p* value of less than 0.05 was considered to be statistically significant.

## Results

### Characteristics of the study population

The final study consisted of 1243 men, 833 idiopathic infertile patients and 410 fertile controls. All subjects were Han Chinese, with a mean (±SD) age of 29.38 (±4.15) years. Eight hundred and thirty three men were idiopathic infertile men, 191 men (22.9%; Case Group I) with normal semen quality and 642 men (77.1%; Case Group II) with abnormal semen quality according to WHO standard. In addition, patients in the Case Group II were further divided into four subgroups: Subgroup I: patients with semen volume < 2 ml (n = 122); Subgroup II: subjects with sperm concentration <20 × 10^6^/ml (n = 340); Subgroup III: patients with sperm number per ejaculum <40 × 10^6^/ml (n = 311); and Subgroup IV: patients with sperm motility <50% motile sperm (n = 611). Demographic categories by fertility and semen quality are described in Table 1. Infertile males were younger, had a lower BMI and were more likely to be former drinkers than the fertile controls (Table 1). All variables were further adjusted for any residue confounding effect in later multivariate logistic regression analyses.

**Table 1:** Characteristics of the study population (n = 1243).

Characteristic	Control (n = 410)^a^	Case Group I (n = 191)^b^	Case Group II (n = 642)^c^	Subgroup I (n = 122)^d^	Subgroup II (n = 340)^e^	Subgroup III (n = 311)^f^	Subgroup IV (n = 611)^g^
Age (mean ± SD)	29.90 ± 3.61	29.19 ± 3.91	29.11 ± 4.51	29.39 ± 4.86	29.15 ± 4.54	29.11 ± 4.59	29.13 ± 4.45
BMI (mean ± SD)^h^	23.77 ± 2.94	23.23 ± 2.92	23.27 ± 3.11	23.48 ± 3.13	23.14 ± 3.11	23.12 ± 3.12	23.26 ± 3.14
Smoking status [n (%)]							
Never smoker	186 (45.4)	84 (44.0)	305 (47.5)	48 (39.3)	142 (41.8)	127 (40.8)	294 (48.1)
Ever smoker	224 (54.6)	107 (56.0)	337 (52.5)	74 (60.7)	198 (58.2)	184 (59.2)	317 (51.9)
Current smoker	199 (48.5)	90 (47.1)	288 (44.9)	66 (54.1)	169 (49.7)	155 (49.8)	268 (43.9)
Former smoker	25 (6.1)	17 (8.9)	49 (7.6)	8 (6.6)	29 (8.5)	29 (9.3)	49 (8.0)
Pack-years (mean ± SD)^f^	4.58 ± 4.70	5.28 ± 4.15	5.33 ± 5.80	5.92 ± 7.79	5.30 ± 6.04	5.43 ± 6.23	5.25 ± 5.81
Drinking status [n (%)]							
Never drinker	187 (45.6)	99 (51.8)	290 (45.2)	56 (45.9)	148 (43.8)	137 (44.1)	274 (44.8)
Ever drinker	223 (54.4)	92 (48.2)	387 (60.3)	66 (54.1)	192 (56.5)	174 (55.9)	337 (55.2)
Current drinker	211 (51.5)	74 (38.7)	292 (45.5)	53 (43.3)	163 (47.9)	147 (47.3)	280 (45.8)
Former drinker	12 (2.9)	18 (9.4)	95 (14.8)	13 (10.7)	29 (8.5)	27 (8.7)	57 (9.3)

SD, standard deviation; BMI, body mass index.

^a^Proven fertility men with semen volume ≥ 2 ml, sperm concentration ≥20 × 10^6^/ml, sperm number per ejaculum ≥40 × 10^6^/ml and sperm motility ≥50% motile sperm.

^b^Idiopathic infertile men with all four semen parameters at or above the WHO reference value.

^c^Idiopathic infertile men with at least one of the abnormal semen parameters (semen volume, sperm concentration, sperm number per ejaculum and sperm motility).

^d^Idiopathic infertile men with semen volume < 2 ml.

^e^Idiopathic infertile men with sperm concentration <20 × 10^6^/ml.

^f^Idiopathic infertile men with sperm number per ejaculum <40 × 10^6^/ml.

^g^Idiopathic infertile men with sperm motility <50% motile sperm.

^h^BMI: kg/m^2^.

### Association between genotype distributions of four *DNA methyltransferases* and semen quality

The genotype distributions of the four *DNMT* genes among the cases and controls are shown in Tables 2 and 3. As shown in Table 2, we initially divided the cases into the Case Group I and Case Group II according to semen parameters. When the *DNMT1* CC genotype was used as the reference group, the CA genotype was significantly associated with increased risk for male infertility in Case Group I (adjusted OR, 1.50, 95% CI = 1.00–2.25, *P* = 0.048) and Case Group II (adjusted OR, 1.40, 95% CI = 1.05–1.86, *P* = 0.023). Under the recessive model of inheritance, the *DNMT1* AA genotype was associated with significantly decreased risk for male infertility with abnormal semen parameters (adjusted OR, 0.68, 95% CI = 0.50–0.92, *P* = 0.012), compared with other genotypes. In addition, compared to the *DNMT3B* CC genotype, the association between the CT genotype and male infertility was not significantly different (adjusted OR, 1.25, 95% CI = 0.85–1.83, *P* = 0.255), but the TT homozygous genotype was associated with significantly decreased risk for male infertility with abnormal semen parameters (adjusted OR, 0.25, 95% CI = 0.06–0.94, *P* = 0.040). Under the recessive model of inheritance, the *DNMT3B* variant homozygote was associated with significantly decreased risk for male infertility with abnormal semen parameters (adjusted OR, 0.24, 95% CI = 0.06–0.90, *P* = 0.034), compared with other genotypes, after adjustment for age, BMI, smoking and drinking status in the multivariate logistic regression analysis (Table 2). In contrast, no significant differences were found for rs1550117 and rs7354779 between the cases and controls.

**Table 2:** Genotype frequencies of DNMTs genetic variants among the cases and controls and their association with male infertility.

SNPs	Genotype	Control (n = 410)^a^	Case Group I (n = 191)^b^			Case Group II (n = 642)^c^		
		n (%)	n (%)	*P* ^d,e^	OR (95% CI)^d,f^	n (%)	*P* ^d,e^	OR (95% CI)^d,f^
rs4804490	CC	144 (35.3)	55 (29.3)		1.00 (reference)	204 (31.9)		1.00 (reference)
	CA	166 (40.7)	96 (51.1)	**0.048**	**1.50 (1.00–2.25)**	324 (50.6)	**0.023**	**1.40 (1.05–1.86)**
	AA	98 (24.0)	37 (19.7)	0.985	1.00 (0.61–1.63)	112 (17.5)	0.281	0.82 (0.58–1.17)
	C-allele carriers	310 (76.0)	151 (80.3)		1.00 (reference)	528 (82.5)		1.00 (reference)
	AA	98 (24.0)	37 (19.7)	0.294	0.79 (0.52–1.22)	112 (17.5)	**0.012**	**0.68 (0.50–0.92)**
rs1550117	GG	263 (64.8)	115 (60.2)		1.00 (reference)	405 (64.0)		1.00 (reference)
	GA	122 (30.0)	70 (36.6)	0.163	1.30 (0.90–1.89)	201 (31.8)	0.829	1.03 (0.78–1.36)
	AA	21 (5.2)	6 (3.1)	0.452	0.69 (0.27–1.80)	27 (4.3)	0.476	0.80 (0.44–1.46)
	G-allele carriers	385 (94.8)	185 (96.9)		1.00 (reference)	606 (95.7)		1.00 (reference)
	AA	21 (5.2)	6 (3.1)	0.315	0.62 (0.24–1.58)	27 (4.3)	0.457	0.80 (0.44–1.44)
rs2424909	CC	344 (86.4)	160 (83.8)		1.00 (reference)	537 (84.8)		1.00 (reference)
	CT	46 (11.6)	30 (15.7)	0.246	1.35 (0.81–2.23)	93 (14.7)	0.255	1.25 (0.85–1.83)
	TT	8 (2.0)	1 (0.5)	0.242	0.29 (0.04–2.32)	3 (0.5)	**0.040**	**0.25 (0.06–0.94)**
	C-allele carriers	390 (98.0)	190 (99.5)		1.00 (reference)	630 (99.5)		1.00 (reference)
	TT	8 (2.0)	1 (0.5)	0.229	0.28 (0.03–2.25)	3 (0.5)	**0.034**	**0.24 (0.06–0.90)**
rs7354779	TT	384 (93.7)	177 (95.2)		1.00 (reference)	597 (94.1)		1.00 (reference)
	TC	24 (5.9)	9 (4.8)	0.555	0.79 (0.35–1.75)	36 (5.7)	0.978	0.99 (0.58–1.70)
	CC	2 (0.5)	0 (0)	—	—	2 (0.3)	0.673	0.65 (0.09–4.73)
	T-allele carriers	408 (99.5)	186 (100)		1.00 (reference)	633 (99.7)		1.00 (reference)
	CC	2 (0.5)	0 (0)	0.593	0.88 (0.55–1.41)	2 (0.3)	0.675	0.65 (0.09–4.74)

SNPs, single-nucleotide polymorphisms. OR, odds ratios; CI, confidence interval.

^a^Proven fertility men with semen volume ≥ 2 ml, sperm concentration ≥20 × 10^6^/ml, sperm number per ejaculum ≥40 × 10^6^/ml and sperm motility ≥50% motile sperm.

^b^Idiopathic infertile men with all four semen parameters at or above the WHO reference value.

^c^Idiopathic infertile men with at least one of the abnormal semen parameters (semen volume, sperm concentration, sperm number per ejaculum and sperm motility).

^d^Adjusted for age, BMI, smoking status and drinking status.

^e^Two-sided χ^2^ test for genotype distributions between cases and controls.

^f^ORs were obtained from multivariate logistic regression analysis.

**Table 3:** Genotype frequencies of *DNMTs* genetic variants among the cases and controls and their association with male infertility.

SNPs	Genotype	Control (n = 410)^a^	Subgroup I (n = 122)^b,f^			Subgroup II (n = 340)^c,f^			Subgroup III (n = 311)^d,f^			Subgroup IV (n = 611)^e,f^		
		n (%)	n (%)	*P* ^g,h^	OR (95% CI)^g,i^	n (%)	*P* ^g,h^	OR (95% CI)^g,i^	n (%)	*P* ^g,h^	OR (95% CI)^g,i^	n (%)	*P* ^g,h^	OR (95% CI)^g,i^
rs4804490	CC	144 (35.3)	42 (34.7)		1.00 (reference)	108 (31.9)		1.00 (reference)	102 (32.9)		1.00 (reference)	193 (31.7)		1.00 (reference)
	CA	166 (40.7)	55 (45.4)	0.525	1.16 (0.73–1.84)	170 (50.1)	**0.037**	**1.43 (1.02–1.99)**	151 (48.7)	0.084	1.35 (0.96–1.90)	309 (50.7)	**0.023**	**1.40 (1.05–1.86)**
	AA	98 (24.0)	24 (19.8)	0.629	0.87 (0.49–1.54)	61 (18.0)	0.606	0.90 (0.59–1.36)	57 (18.4)	0.624	0.90 (0.59–1.38)	107 (17.6)	0.287	0.83 (0.58–1.18)
	C-allele carriers	310 (76.0)	97 (80.2)		1.00 (reference)	278 (82.0)		1.00 (reference)	253 (81.6)		1.00 (reference)	502 (82.4)		1.00 (reference)
	AA	98 (24.0)	24 (19.8)	0.357	0.79 (0.48–1.31)	61 (18.0)	0.066	0.71 (0.50–1.02)	57 (18.4)	0.099	0.73 (0.51–1.06)	107 (17.6)	**0.014**	**0.68 (0.50–0.92)**
rs1550117	GG	263 (64.8)	82 (67.2)		1.00 (reference)	215 (64.2)		1.00 (reference)	193 (63.1)		1.00 (reference)	381 (63.3)		1.00 (reference)
	GA	122 (30.0)	34 (27.9)	0.600	0.89 (0.56–1.40)	104 (31.0)	0.964	1.01 (0.73–1.39)	100 (32.7)	0.597	1.09 (0.79–1.52)	196 (32.6)	0.645	1.07 (0.81–1.41)
	AA	21 (5.2)	6 (4.9)	0.842	0.91 (0.35–2.35)	16 (4.8)	0.806	0.92 (0.46–1.83)	13 (4.2)	0.618	0.83 (0.40–1.73)	25 (4.2)	0.488	0.81 (0.44–1.48)
	G-allele carriers	385 (94.8)	116 (95.1)		1.00 (reference)	319 (93.2)		1.00 (reference)	293 (95.8)		1.00 (reference)	577 (95.8)		1.00 (reference)
	AA	21 (5.2)	6 (4.9)	0.911	0.95 (0.37–2.42)	16 (4.8)	0.770	0.90 (0.46–1.78)	13 (4.2)	0.527	0.79 (0.38–1.63)	25 (4.2)	0.453	0.79 (0.43–1.45)
rs2424909	CC	344 (86.4)	102 (85.0)		1.00 (reference)	290 (86.6)		1.00 (reference)	264 (86.3)		1.00 (reference)	509 (84.6)		1.00 (reference)
	CT	46 (11.6)	18 (15.0)	0.383	1.30 (0.72–2.36)	43 (12.8)	0.792	1.06 (0.68–1.67)	40 (13.1)	0.703	1.09 (0.69–1.73)	90 (15.0)	0.220	1.27 (0.87–1.87)
	TT	8 (2.0)	0 (0.0)	0.980	1.00 (0.82–1.23)	2 (0.6)	0.145	0.31 (0.06–1.50)	2 (0.7)	0.185	0.35 (0.07–1.66)	3 (0.5)	**0.046**	**0.26 (0.07–0.98)**
	C-allele carriers	390 (98.0)	120 (100.0)		1.00 (reference)	333 (99.4)		1.00 (reference)	304 (99.3)		1.00 (reference)	599 (99.5)		1.00 (reference)
	TT	8 (2.0)	0 (0.0)	0.984	1.00 (0.82–1.23)	2 (0.6)	0.138	0.31 (0.06–1.47)	2 (0.7)	0.175	0.34 (0.07–1.62)	3 (0.5)	**0.040**	**0.25 (0.06–0.94)**
rs7354779	TT	384 (93.7)	110 (91.7)		1.00 (reference)	311 (92.6)		1.00 (reference)	282 (91.9)		1.00 (reference)	567 (93.9)		1.00 (reference)
	TC	24 (5.9)	9 (7.5)	0.529	1.29 (0.58–2.87)	24 (7.1)	0.443	1.26 (0.70–2.27)	24 (7.8)	0.273	1.39 (0.77–2.52)	36 (6.0)	0.862	1.05 (0.61–1.79)
	CC	2 (0.5)	1 (0.8)	0.640	1.79 (0.16–20.28)	1 (0.3)	0.632	0.55 (0.05–6.52)	1 (0.3)	0.701	0.61 (0.05–7.40)	1 (0.2)	0.324	0.29 (0.02–3.44)
	T-allele carriers	408 (99.5)	119 (99.2)		1.00 (reference)	335 (99.7)		1.00 (reference)	306 (99.7)		1.00 (reference)	603 (99.8)		1.00 (reference)
	CC	2 (0.5)	1 (0.8)	0.675	1.68 (0.15–19.13)	1 (0.3)	0.626	0.54 (0.04–6.46)	1 (0.3)	0.690	0.60 (0.05–7.27)	1 (0.2)	0.325	0.29 (0.02–3.45)

SNPs, single-nucleotide polymorphisms. OR, odds ratios; CI, confidence interval.

^a^Proven fertility men with semen volume ≥ 2 ml, sperm concentration ≥20 × 10^6^/ml, sperm number per ejaculum ≥40 × 10^6^/ml and sperm motility ≥50% motile sperm.

^b^Idiopathic infertile men with semen volume, 2 ml.

^c^Idiopathic infertile men with sperm concentration, 20 × 10^6^/ml.

^d^Idiopathic infertile men with sperm number per ejaculum, 40 × 10^6^/ml.

^e^Idiopathic infertile men with sperm motility, 50% motile sperm.

^f^The semen parameter categories were not mutually exclusive; a subject may contribute data to more than one category.

^g^Adjusted for age, BMI, smoking status and drinking status.

^h^Two-sided χ^2^ test for genotype distributions between cases and controls.

^I^ORs were obtained from multivariate logistic regression analysis.

In order to further assess the possibility of an association between the four *DNMTs* genetic variants and a particular aspect of semen parameters, the patients in case Group II were further stratified into four subgroups on the basis of semen volume, sperm concentration, sperm number per ejaculum or sperm motility as described in the section of statistical analysis. As illustrated in Table 3, subjects in Subgroup II and Subgroup IV had a significantly increased frequency of the heterozygous *DNMT1* CA genotype compared with fertile controls (adjusted OR, 1.43, 95% CI = 1.02–1.99, *P* = 0.037 for Subgroup II; adjusted OR, 1.40, 95% CI = 1.05–1.86, *P* = 0.023 for Subgroup IV). Under the recessive model of inheritance, subjects in Subgroup IV had a significantly decreased frequency of the *DNMT1* variant homozygote compared with other genotypes (adjusted OR, 0.68, 95% CI = 0.50–0.92, *P* = 0.014). When the *DNMT3B* CC genotype was used as the reference group, the carrier of TT genotype in Subgroup IV had a significantly decreased risk of male infertility (adjusted OR, 0.26, 95% CI = 0.07–0.98, *P* = 0.046). The *DNMT3B* variant homozygote had a significantly decreased risk of male infertility in the Subgroup IV (adjusted OR, 0.25, 95% CI = 0.06–0.94, *P* = 0.040) compared with other genotypes (Table 3). In contrast, no significant differences were found for *DNMT3A* (rs1550117) and *DNMT3L* (rs7354779) between the cases and controls (Table 3).

### Effects of the variant rs2424909 and rs4804490 on the binding of transcription factors

According to AliBaba2 programs, with the transition of “C” to “T” of the variant *DNMT3B* rs2424909 allele, new binding sites for transcription factors (Figure S1) were generated. Figure S2 indicated that the transition of “C” to “A” of the variant *DNMT*1 rs4804490 allele generated new binding sites for transcription factors. Figures S3 and S4 showed that the transition of “A” to “G” of the variant *DNMT3A* rs1550117 allele and “C” to “T” of the variant *DNMT3L* rs7354779 generated new binding sites for transcription factors.

## Discussion

Several studies have revealed the close relationship of DNMTs with the male reproductive system, and potential associations with human infertility. Although it has been identified that DNMTs play critical roles in spermatogenesis and male fertility, a genetic study of four *DNMT*s in idiopathic male infertility has not been performed yet.

This case-control study analyzed *DNMTs* polymorphisms to define their association with semen parameters and male infertility. We have demonstrated a significantly increased risk of idiopathic male infertility with abnormal semen parameters in association with heterozygote of variant rs4804490, and a decreased risk of idiopathic infertility with abnormal semen parameters in association with variant homozygote of variant rs2424909 (*P* = 0.023 and 0.040, respectively). In addition, under the recessive model of inheritance, the AA genotype of variant rs4804490 and TT genotype of variant rs2424909 were significantly associated with decreased risk for male infertility with abnormal semen parameters. Among men with normal semen parameters, there were no significant differences in risks associated with these genotypes. Furthermore, no significant associations with risk of idiopathic infertility associated with either normal or abnormal semen parameters were found for the rs1550117 and rs7354779 variants. A recent study by Huang *et al*. investigated the association between *DNMT3L* polymorphisms and male infertility with azoospermia^33^. They found that the presence of rs2070565 allele A increased the risk of azoospermia in males. Additionally, they did not report any significant difference for rs7354779 between the infertile patients and fertile controls, which similar to our findings. These results suggest that different variants in the genes involved in epigenetic pathways may have different relationships with idiopathic male infertility and men with these variants have an increased or increased risk of abnormal semen parameters associated with male infertility.

Genetic disruption of both *DNMT1* and *DNMT3B* nearly eliminated methyltransferase activity, and reduced genomic DNA methylation by greater than 95%^34^. These marked changes resulted in demethylation of repeated sequences, loss of insulin-like growth factor II (*IGF2*) imprinting, abrogation of silencing of the tumor suppressor gene p16, and growth suppression^34^. Rhee and colleagues demonstrated that these two enzymes cooperatively maintain DNA methylation and gene silencing in human cancer cells^34^. The precise mechanism of the variant rs4804490 and rs2424909 in male infertility remains unclear, as there is no direct functional data available. A single-base substitution may change the binding of transcription factors and consequently influence their function. We therefore raised hypotheses that these variations may be associated with changes of transcription factors. Changes of the binding of transcription factors were observed with both of the variations. Variants of *DNMT1* and *DNMT3B* are risk factors for idiopathic male infertility.

In conclusion, our study demonstrates that some representative variants of the DNMT genes may modulate the risk of male infertility associated with abnormal semen parameters. Confirmation of similar findings in larger study groups would be needed. In summary, in this case-control study, we found that the carrier of *DNMT1* CA genotype had a significantly increased risk of idiopathic male infertility compared with *DNMT1* CC genotype carriers, although no significant difference was found in *DNMT3A* and *DNMT3L*. Our results support the hypothesis that *DNMT1* polymorphism may be associated with an increased risk of idiopathic male infertility in a Han-Chinese population. These SNPs may alter a transcription factor binding site in the alternative promoter, and the variant genotype (s) may be in linkage disequilibrium with other untyped susceptibility loci. Moreover, the variants may alter the binding of a regulatory miRNA or contribute to the differential expression of alternatively spliced *DNMT3B* variants.

As shown in Fig. S1, the rs2424909 C > T variation creates a binding site for transcription factors GR, and deletes two binding sites for ADR1 and AP-2alph. GR is a ligand-activated transcription factor. In addition to activating enhancers harboring glucocorticoid response elements (GREs), it also inhibits the actions of other transcription factors, including AP1 and NF-κB. In ERα-positive breast cancer, GR expression has been associated with good clinical outcomes^35, 36^. AP-1 transcription factor, known to play a vital role in cell proliferation and neuronal activation, is also involved in cell apoptosis in response to stress, lack of survival signals or DNA damaging agents^37^. Nuclear factor-κB (NF-κB) transcription factor regulates a wide array of genes mediating numerous cellular processes such as proliferation, differentiation, motility and survival^38^. In Fig. S2, rs4804490 C > A variation creates two binding sites for transcription factors NF-1 and CTF, deleting two binding sites for c-Jun and Sp1. The CAAT box-binding transcription factor/nuclear factor-1 (NF1, also called CTF/NF1) consists of widely expressed transcription factors, comprising NF1-A, NF1-B, NF1-C and NF1-X subtypes^39, 40^. Transcription factor Specificity protein 1 (Sp1) plays a role in promoting oncogenes required for tumor survival, metastasis and progression. c-Jun, a component of the transcription factor AP-1, regulates gene expression and cell function.

At present, there is no evidence that these transcription factors interact with DNMTs to control their DNA binding specificity. We suggest that the genetic variants *DNMT3B* and *DNMT1* could regulate gene expression by the creation or deletion of transcription factors and influence the sperm maturation process and male fertility. Microarray analysis and real time quantitative PCR has shown that the differential gene expression of *DNMT1* between various forms of testicular cancer was consistent^41^. Adiga *et al*. documented that *DNMT3B* is differentially expressed^42^ in the preleptotene/zygotene and pachytene spermatocytes from fertile and infertile men. Future mechanistic studies about functional significance of the variation at *DNMT1* and *DNMT3B* will help fully flesh out the relationship between polymorphism of DNMTs, abnormal DNA methylation patterns, and spermatogenic failure.

## Electronic supplementary material

Supplementary Information
