# Supplementary material for: Idiopathic male infertility and polymorphisms in the DNA methyltransferase genes involved in epigenetic marking
Source: Sci Rep. 2017 Sep 11;7:11219. doi: 10.1038/s41598-017-11636-9 (PMC5593912; doi:10.1038/s41598-017-11636-9)
Supplement: Supplementary file 1 — Supplementary Information [file 41598_2017_11636_MOESM1_ESM.pdf]

**Title page**

# Idiopathic male infertility and polymorphisms in the DNA methyltransferase genes involved in epigenetic marking

Qiuqin Tang[1, †], Yiqiu Chen[2, 3, †], Wei Wu[2, 3, 4, *], Hongjuan Ding[1], Yankai Xia[2, 3, *], Daozhen Chen[4], Xinru Wang[2, 3]

[1] State Key Laboratory of Reproductive Medicine, Department of Obstetrics, Nanjing Maternity and Child Health Hospital, Obstetrics and Gynecology Hospital Affiliated to Nanjing Medical University, Nanjing 210004, China

[2] State Key Laboratory of Reproductive Medicine, Institute of Applied Toxicology, School of Public Health, Nanjing Medical University, Nanjing 211166, China

[3] Key Laboratory of Modern Toxicology of Ministry of Education, Nanjing Medical University, Nanjing 211166, China

[4] Clinical Laboratory, Wuxi Maternity and Child Health Care Hospital Affiliated to Nanjing Medical University, Wuxi 214002, China

[†] These authors contributed equally to the study and they should be regarded as joint first authors.

[*]To whom correspondence should be addressed:

Wei Wu Ph.D. or Yankai Xia Ph.D.

State Key Laboratory of Reproductive Medicine, Institute of Toxicology,

Nanjing Medical University, 101 Longmian Avenue, Nanjing 211166, China.

Tel: +86-25-86868425 Fax: +86-25-86868499

Email: wwu@njmu.edu.cn; yankaixia@njmu.edu.cn

## Figure legends

**Figure S1. A predicted effect of the variant rs2424909 on transcription factor-binding site.** Comparison between C allele (A) and T allele (B) of rs2424909 for the putative transcriptional factor-binding site.

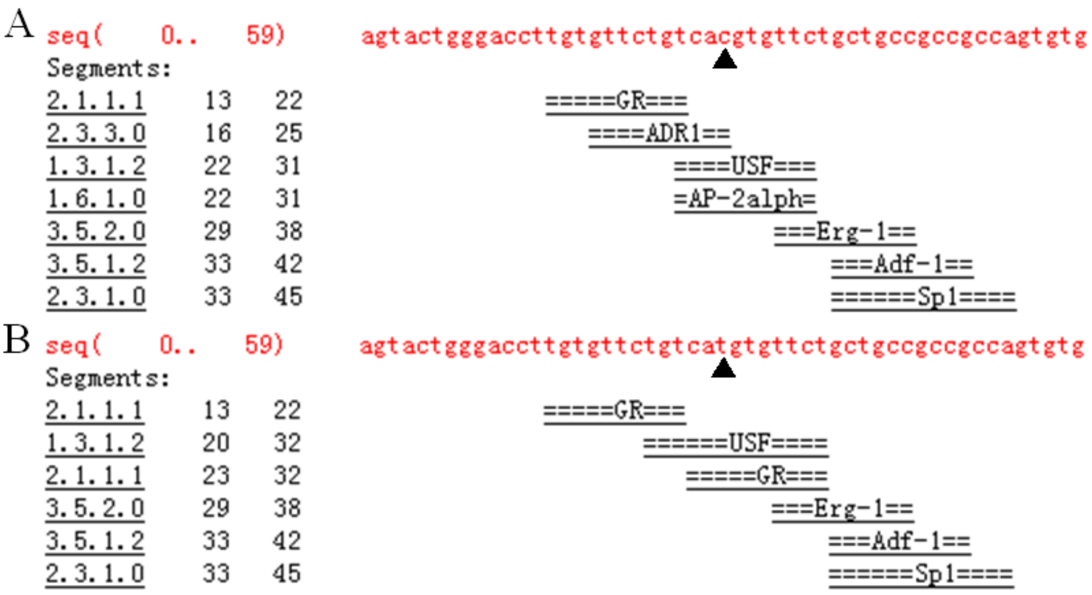


**Figure S2. A predicted effect of the variant rs4804490 on transcription factor-binding site.** Comparison between C allele (A) and A allele (B) of rs4804490 for the putative transcriptional factor-binding site.

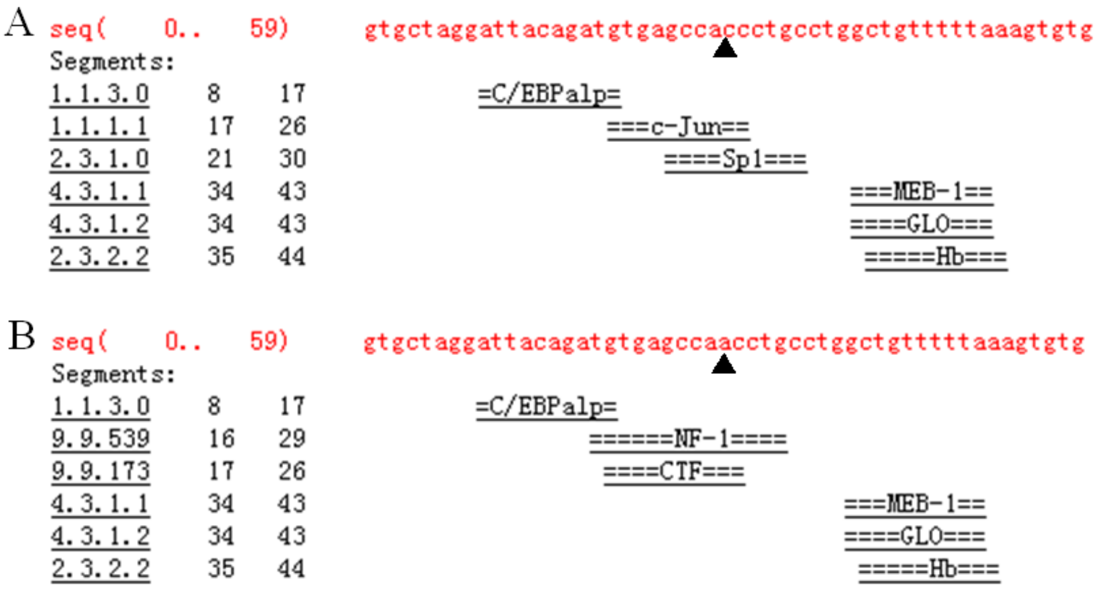

**Figure S3. A predicted effect of the variant rs1550117 on transcription factor-binding site.** Comparison between A allele (A) and G allele (B) of rs1550117 for the putative transcriptional factor-binding site.

A
```
seq(     0..    59)   aattccaccagcacagccactcactatgtgctcatctcactcctccagcag
                                               ▲
Segments:
2.3.1.0   10   19               ====Sp1===
1.1.3.0   24   33                             =C/EBPalp=
2.3.1.0   36   48                                         ======Sp1====
1.2.2.0   44   53                                                 ====MRF4==
```

B
```
seq(     0..    59)   aattccaccagcacagccactcactgtgtgctcatctcactcctccagcag
                                               ▲
Segments:
2.3.1.0   10   19               ====Sp1===
2.3.1.0   16   25                     ====Sp1===
2.1.1.1   21   30                          =====GR===
1.1.3.0   24   33                             =C/EBPalp=
2.3.1.0   36   48                                         ======Sp1====
1.2.2.0   44   53                                                 ====MRF4==
```

**Figure S4. A predicted effect of the variant rs7354779 on transcription factor-binding site.** Comparison between C allele (A) and T allele (B) of rs7354779 for the putative transcriptional factor-binding site.

A
```
seq(     0..    59)   tccacgaacatccagaagaagggcccggggctgcctggcttgggccgtgcg
                                               ▲
Segments:
1.6.1.0   20   29                          =AP-2alph=
2.3.1.0   20   31                          =====Sp1====
2.3.1.0   41   50                                               ====Sp1===
```

B
```
seq(     0..    59)   tccacgaacatccagaagaagggcctggggctgcctggcttgggccgtgcg
                                               ▲
Segments:
2.3.1.0   15   29                     =======Sp1=====
1.6.1.0   20   30                          =AP-2alphaA
2.3.1.0   21   33                           ======Sp1====
2.3.1.0   41   50                                               ====Sp1===
```
